# The Subtilisin-Like Protease Bcser2 Affects the Sclerotial Formation, Conidiation and Virulence of *Botrytis cinerea*

**DOI:** 10.3390/ijms21020603

**Published:** 2020-01-17

**Authors:** Xinqiang Liu, Jiatao Xie, Yanping Fu, Daohong Jiang, Tao Chen, Jiasen Cheng

**Affiliations:** 1 State Key Laboratory of Agricultural Microbiology, Huazhong Agricultural University, Wuhan 430070, China; liuxinqang@163.com (X.L.); jiataoxie@mail.hzau.edu.cn (J.X.); daohongjiang@mail.hzau.edu.cn (D.J.); taochen@mail.hzau.edu.cn (T.C.); 2 The Provincial Key Lab of Plant Pathology of Hubei Province, College of Plant Science and Technology, Huazhong Agricultural University, Wuhan 430070, China; yanpingfu@mail.hzau.edu.cn

**Keywords:** *Botrytis cinerea*, subtilisin-like serine proteases, *Bcser2*, sclerotial formation, conidiation, virulence

## Abstract

*Botrytis cinerea*, a ubiquitous necrotrophic plant-pathogenic fungus, is responsible for grey mold and rot disease in a very wide range of plant species. Subtilisin-like proteases (or subtilases) are a very diverse family of serine proteases present in many organisms and are reported to have a broad spectrum of biological functions. Here, we identified two genes encoding subtilisin-like proteases (*Bcser1* and *Bcser2*) in the genome of *B. cinerea,* both of which contain an inhibitor I9 domain and a peptidase S8 domain. The expression levels of *Bcser1* and *Bcser2* increased during the sclerotial forming stage, as well as during a later stage of hyphal infection on *Arabidopsis thaliana* leaves, but the up-regulation of *Bcser1* was significantly higher than that of *Bcser2*. Interestingly, deletion of *Bcser1* had no effect on the fungal development or virulence of *B. cinerea.* However, deletion of *Bcser2* or double deletion of *Bcser1* and *Bcser2* severely impaired the hyphal growth, sclerotial formation and conidiation of *B. cinerea*. We also found that *∆Bcser2* and *∆Bcser1*/*2* could not form complete infection cushions and then lost the ability to infect intact plant leaves of *Arabidopsis* and tomato but could infect wounded plant tissues. Taken together, our results indicate that the subtilisin-like protease *Bcser2* is crucial for the sclerotial formation, conidiation, and virulence of *B. cinerea*.

## 1. Introduction

*Botrytis cinerea*, the causal agent of grey mold, is considered a typical necrotrophic plant-pathogenic fungus and causes significant losses in more than 1000 plant species worldwide [1,2,3]. Because of its great economic implications and scientific significance, *B. cinerea* was considered as the second most important plant-pathogenic fungus in a paper published on the journal molecular plant pathology in 2012 [1] and has become a model fungal system for the study of necrotrophic pathogens [4,5,6]. *B. cinerea* produces different structures, including sclerotia, macroconidia, microconidia, and fruiting bodies called apothecia, in its lifecycle [7]. Sclerotia are loosely described as nutrient-rich, morphologically variable and multihyphal structures [8]. The melanized sclerotia plays crucial roles in the lifecycle of *B. cinerea* and may either serve as asexual survival structure for local colonization or as the maternal parent in sexual reproduction to initiate the sexual cycle, including the production of apothecia [4,9]. The sclerotia also can be a source of conidia that result in infection of plants, which is more common in nature because *B. cinerea* is dispersed predominantly via conidia [4]. Disease control of *B. cinerea* mostly relies on chemical fungicides, but there is increasing concern about the evolution of fungicide resistance in *B. cinerea* populations [10,11].

Fungi secrete abundant depolymerizing enzymes to digest complex substrates in their environment for nutritional acquisition [12]. Extracellular proteases are produced by many plant-pathogenic fungi and are commonly involved in the degradation of the host extracellular matrix, facilitating invasion and colonization [13]. Based on the catalytic mechanism, proteases are now divided into seven classes: serine, metallo-, aspartic, cysteine, threonine, glutamic, and asparagine proteases, with other proteases that are unknown or mixed [14]. Serine proteases are a class of peptidases that are extremely widely distributed in all kingdoms of cellular life as well as many viral genome and use a nucleophilic serine residue in the enzyme active site to cleave peptides [15]. Subtilisins are a kind of serine protease that was first discovered in *Bacillus subtilis* and named after the organism in which it was observed for the first time [16]. A common feature of subtilisins is their catalytic triad that consists of three residues, D, H, and S, in the active site. The catalytic triad is highly conserved among all known subtilisins [17].

Subtilisin-like proteases (also known as subtilases) compose a subfamily in the clan SB of serine peptidases, family S8A, according to the *MEROPS* database [18,19]. Many recent studies have shown that subtilases play important roles in development and virulence of pathogenic fungi [20]. For example, disruption of the subtilase *Cerevisin* affects microsclerotia formation and virulence in *Verticillium dahliae*. Furthermore, the results showed that *Cerevisin* is involved in secretion of low molecular weight (14–25 kDa) proteins and controlling multiple processes of development and metabolism [21]. In *Pseudogymnoascus destructans*, the fungus responsible for white nose syndrome (WNS) in bats, a dominant subtilase PdSP1 was identified and considered to be potentially involved in the WNS host–pathogen interaction [12]. Deletion of a subtilisin-like protease encoded gene *prb1* results in lower level of sporulation and aerial hyphae, and a remarkable reduction in virulence in the chestnut blight fungus *Cryphonectria parasitica.* The result also showed that loss of function of *prb1* affects the accumulation of autophagic bodies [22]. This result was consistent with a previous study, which found that disruption of *prb1* in *Saccharomyces cerevisiae* results in the accumulation of autophagic bodies in vacuoles and reduction in sporulation [23,24]. In *Magnaporthe grisea*, a subtilase encoding gene *spm1* has been shown to be involved in sporulation, appressorium formation and pathogenicity and its function is also related to autophagy [25]. In summary, subtilases are involved in the development and virulence in a variety of pathogenic fungi.

In *B. cinerea*, two genes (*Bcser1* and *Bcser2*) encoding subtilisin-like serine proteases showed a significant level of expression during the colonization of sunflower cotyledons [26]. However, the gene functions of *Bcser1* and *Bcser2* remain to be elucidated. To date, there is no information on the role of subtilisin-like proteases in *B. cinerea*. To better understand the biological function of subtilisin-like proteases in *B. cinerea*, we constructed and characterized mutants of *Bcser1* and *Bcser2*. We report here that deletion of *Bcser1* had no obvious effect on sclerotial formation, sporulation or virulence of *B. cinerea*. However, deletion of *Bcser2* or double deletion of *Bcser1* and *Bcser2* resulted in severely impaired sclerotial formation, sporulation and virulence. We also found that *∆Bcser2* and *∆Bcser1*/*2* could not form complete infection cushions during infection. Our results suggest that *Bcser2* is essential for the development and pathogenesis of *B. cinerea*.

## 2. Results

### 2.1. Analysis of Bcser1 and Bcser2 in B. cinerea

**Figure 1:**
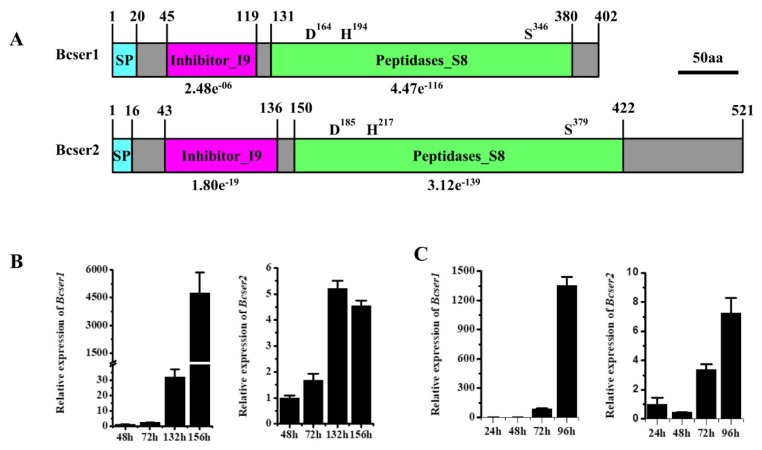
Analysis of the subtilisin-like proteins *Bcser1* and *Bcser2*. (**A**) Analysis of putative conserved domains encoded by *Bcser1* and *Bcser2*. (**B**) Relative levels of transcript accumulation of *Bcser1* and *Bcser2* were determined by qRT-PCR when cultivated on PDA medium at 20 °C. The relative levels of transcripts were calculated using the comparative Ct method. The levels of *BcActin* transcript of *B. cinerea* were used to normalize the expression levels. Values are the means of three independent trials. Bars indicate ±SE. (**C**) Relative levels of transcript accumulation of *Bcser1* and *Bcser2* were determined by qRT-PCR in inoculated *Arabidopsis* plants at 20 °C. The levels of *BcActin* transcript of *B. cinerea* were used to normalize the expression levels.

Two genes that encoded subtilisin-like serine proteases, *Bcser1* (Gene ID: Bcin10g02530) and *Bcser2* (Gene ID: Bcin08g02990), were found in *B. cinerea* by using the program BlastP [27]. The open reading frame of *Bcser1* is 1206 bp in length and is predicted to encode a 402 aa protein with a predicted N-terminal SP (1–20 aa) [28]. *Bcser1* is predicted to have an inhibitor I9 (45–119 aa) conserved domain and a peptidase S8 (131–380 aa) conserved domain with a predicted catalytic triad (D164, H194, and S346), suggesting that it may be a subtilisin-like serine protease (Figure 1A). Inhibitor I9 domain of subtilases is an auto-inhibitory domain which can prevent the access of the substrate to the active site and maintain the inactive state of the zymogen. Inhibitor I9 domain also works as an intramolecular chaperone that is transiently required to assist catalytic domain folding [29]. The open reading frame of *Bcser2* is 1563 bp in length and is predicted to encode a 521 aa protein with a predicted N-terminal SP (1–16 aa). *Bcser2* also has an inhibitor I9 (43–136 aa) conserved domain and a peptidase S8 (150–422 aa) conserved domain with a predicted catalytic triad (D185, H217, and S379), suggesting that it may also be a subtilisin-like serine protease (Figure 1A).

### 2.2. Bcser2 Plays Important Roles in Sclerotial Formation and Conidiation

**Figure 2:**
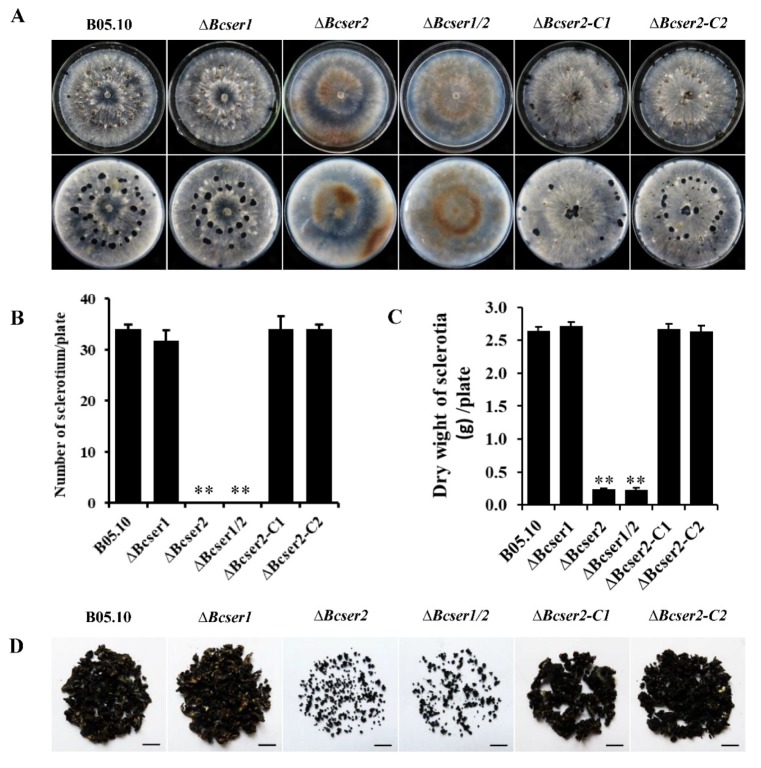
Effects of *Bcser1* and *Bcser2* deletion on sclerotial development of *B. cinerea*. (**A**) Phenotype of deletion mutants *∆Bcser1*, *∆Bcser2* and *∆Bcser1*/*2*, complemented mutants *∆Bcser2-C1* and *∆Bcser2-C2*, and wild-type strain B05.10 grown on potato dextrose agar (PDA) medium at 20 °C in complete darkness. The top row shows the front of the colony and the bottom row shows the back of the colony. Photographs were captured at 15 days post-inoculation (dpi). (**B**) Comparison of sclerotia number in strains growing on a PDA plate at 20 °C for 30 days in complete darkness. (**C**) The dry weight of *∆Bcser2* and *∆Bcser1*/*2* sclerotia grown on carrot cube medium are noticeably reduced compared to the WT and complemented strains. (**D**) Sclerotia yielded by wild-type strain B05.10 and all mutants per flask. Three independent replications were performed for each treatment. Bars indicate ±SE. Statistical significance is indicated in the graph (one-way ANOVA): **, *p* < 0.01.

**Figure 3:**
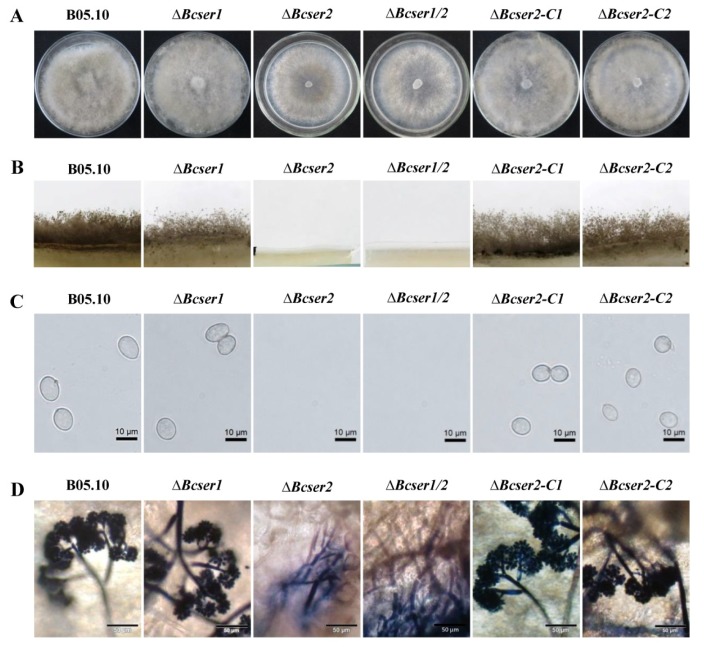
Effects of *Bcser1* and *Bcser2* deletion on conidiophore and conidia development of *B. cinerea*. (**A**) Phenotype of mutants and wild-type strain B05.10 grown on potato dextrose agar (PDA) medium at 20 °C under a 12 h light/dark cycles. Photographs were captured at 14 days post-inoculation (dpi). (**B**) Production of conidiophores on PDA medium at 20 °C under a 12 h light/dark cycles. (**C**) Conidiation of deletion mutants *∆Bcser1*, *∆Bcser2* and *∆Bcser1*/*2*, complemented mutants *∆Bcser2-C1* and *∆Bcser2-C2*, and wild-type strain B05.10 grown on PDA medium at 20 °C under continuous light. Scale bar, 10 μm. (**D**) Production of conidiophores on wounded *Arabidopsis* leaves. Scale bar, 50 μm.

qRT-PCR analysis was used to determine the expression patterns of *Bcser1* and *Bcser2* in *B. cinerea* during growth and infection. Results indicated that when B05.10 strain was inoculated on PDA, the transcript levels of both *Bcser1* and *Bcser2* were significantly up-regulated at 132 hpi and 156 hpi, the peak of *Bcser1* gene expression was at 156 hpi, while *Bcser2* was at 136 hpi (Figure 1B). When B05.10 strain was inoculated on leaves of *A. thaliana*, the transcript levels of *Bcser1* and *Bcser2* were significantly up-regulated at 72 hpi and 96 hpi, and the transcript levels of both genes were highest at 96 hpi (Figure 1C). Furthermore, the semi-quantitative RT-PCR results are consistent with these results (Figure S3). The results suggested that *Bcser1* and *Bcser2* may play important roles during sclerotial formation and pathogenesis of *B. cinerea*. To explore the function of *Bcser1* and *Bcser2* in *B. cinerea*, gene deletion mutants were generated using a homologous recombination strategy (Figure S1A). A total of 4, 6, and 6 transformants of *Bcser1*, *Bcser2*, and a double gene deletion were generated and checked by PCR and Southern blot analysis (Figure S1C) to confirm that the targeted gene was replaced by a single copy marker gene. After that, several rounds of single-spore isolation were carried out until the target gene was completely removed from the mutant strains [30]. Isolated mutants were checked via PCR analysis (Figure S1D–F). After that, biological phenotypes such as the sclerotial formation and conidiation of WT, *Bcser1* deletion mutant *∆Bcser1*, *Bcser2* deletion mutant *∆Bcser2*, and double deletion mutant *∆Bcser1*/*2* were compared. The results showed that the deletion of *Bcser1* had no obvious effect on conidiation and sclerotial formation of *B. cinerea* (Figure 2 and Figure 3). Interestingly, *∆Bcser2* and *∆Bcser1*/*2* mutants failed to produce any sclerotia on PDA medium in darkness at 20 °C for 2 weeks, while the WT strain formed many black sclerotia (Figure 2A,B). At the same time, the mutant ∆*Bcser2* and ∆*Bcser1*/*2* showed a slightly slower growth rate on PDA medium compared with the WT strain (Figure S4C). When cultured on sterilized carrot fragments for two months, *∆Bcser2* and *∆Bcser1*/*2* strains formed relatively fewer and much smaller sclerotia (Figure 2C,D, and Figure S5). Furthermore, the mutants ∆*Bcser2* and ∆*Bcser1*/*2* lost the ability to produce conidia on PDA medium (Figure 3A–C). Our results showed that the colony surface of *∆Bcser2* and *∆Bcser1*/*2* are very smooth, however, the colony surface of wild-type and complementary strain are full of aerial hypha (Figure 3A). Moreover, there were no conidiophores were formed in *∆Bcser2* and *∆Bcser1*/*2* mutants, while a large number of conidiophores were formed in the wild-type and complementary strain (Figure 3B). When ∆*Bcser2* and ∆*Bcser1*/*2* mutants were inoculated into *Arabidopsis* leaves, they again could not produce any conidiophores and conidia (Figure 3D). These results indicated that *Bcser2* may be involved in sclerotial formation and conidiation in *B. cinerea*. To confirm the important roles of Bcser2 in sclerotial formation and conidiation in *B. cinerea*, gene in situ complementation mutants were generated using a homologous recombination strategy in mutant ∆*Bcser2*. Single spore isolated complementary strains ∆*Bcser2-C1* and ∆*Bcser2-C2* were checked via PCR and RT-PCR analysis (Figure S2). Our results showed that the *Bcser2* complementary strains ∆*Bcser2-C1* and ∆*Bcser2-C2* restored sclerotial formation and conidiation to the level of WT strain B05.10. These results demonstrated that *Bcser2* plays an important role in sclerotial formation and conidiation in *B. cinerea*, and *Bcser1* seems to not be essential in these processes.

### 2.3. Bcser2 Is Essential for the Full Virulence of B. cinerea

**Figure 4:**
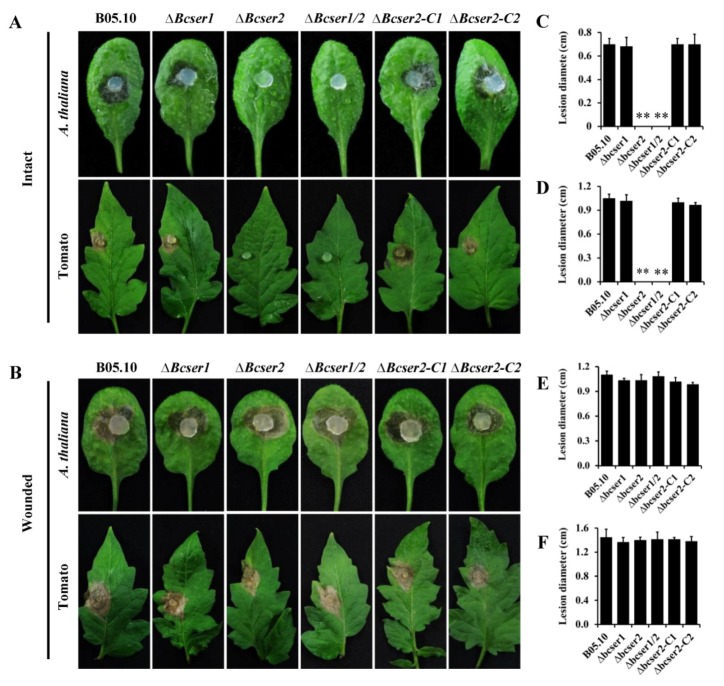
Virulence assays of mutants and wild-type strains on detached *Arabidopsis* and tomato leaves. (**A**) Intact leaves of *Arabidopsis* and tomato were inoculated with mycelial agar plugs (diameter = 3 mm) from the margins of actively growing colonies of deletion mutants *∆Bcser1*, *∆Bcser2,* and *∆Bcser1*/*2*, complemented mutants *∆Bcser2-C1* and *∆Bcser2-C2*, and wild-type strain B05.10 on PDA plates. Photographs were captured at 72 hpi. (**B**) Wounded leaves of *Arabidopsis* and tomato were inoculated with mycelial agar plugs (3 mm diameter) from the margins of actively growing colonies of the indicated strains on PDA plates. Detached leaves were wounded by a sterilized inoculation needle to make three small holes and mycelial agar plugs were inoculated onto the wound. For *Arabidopsis* and tomato, Photographs were captured at 24 hpi and 36 hpi, respectively. (**C**) Virulence on intact *Arabidopsis* leaves was evaluated based on lesion diameters. (**D**) Virulence on intact tomato leaves was evaluated based on lesion diameters. (**E**) Virulence on wounded *Arabidopsis* leaves was evaluated based on lesion diameters. (**F**) Virulence on wounded tomato leaves was evaluated based on lesion diameters. In all experiments, three independent replicates were performed each with 6 leaves from three plants. Values are presented as the means ± SE. Statistical significance is indicated in the graph (one-way ANOVA): **, *p* < 0.01.

To assess the virulence of the mutants of *Bcser1* and *Bcser2*, infection experiments on different plant leaves were performed. After inoculation, the box containing leaves was placed in a chamber at 20 °C with a 16-h photoperiod. The results showed that *∆Bcser2* and *∆Bcser1*/*2* failed to penetrate intact leaves of *Arabidopsis* or tomato, while the *∆Bcser1* mutant, WT strain and the complemented strains *∆Bcser2-C1* and *∆Bcser2-C2* caused serious disease lesions (Figure 4A), suggesting that *Bcser2* is essential for the full virulence of *B. cinerea*. Interestingly, both *∆Bcser2* and *∆Bcser1*/*2* could successfully infect wounded leaves of *Arabidopsis* and tomato. Furthermore, the disease lesions induced by *∆Bcser2* and *∆Bcser1*/*2* mutants on wounded leaves were almost the same as induced by the WT strain (Figure 4B). In addition, virulence assays on *Arabidopsis* also showed that the lesions induced by the wild-type and complementary strain continued to expand at 4 dpi, and the leaf surface is totally invaded and rotted at 7 dpi, but the ∆*Bcser2* and ∆*Bcser1*/*2* mutants still did not produce any lesions at this time point (Figure S6). These results further indicate that *Bcser2* is crucial to the virulence of *B. cinerea*. 

### 2.4. Bcser2 Is Required for Infection Cushion Formation

**Figure 5:**
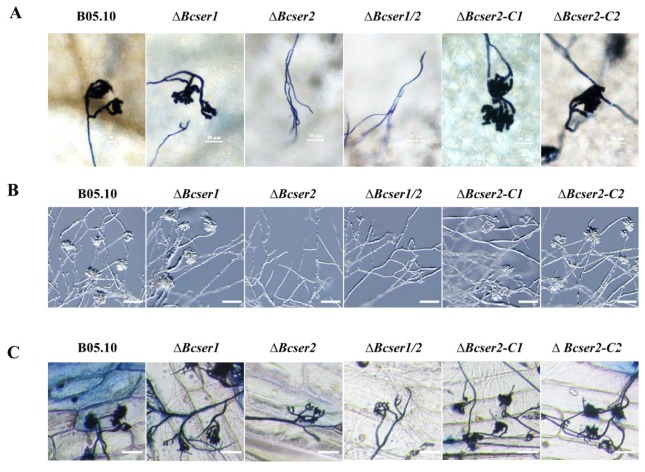
Infection cushions (ICs) formation of *B. cinerea*. (**A**) Microscopic observation of ICs formation of wild-type B05.10 and mutants on *Arabidopsis* leaves after staining with Trypan blue. The mycelial agar plugs (3 mm diameter) of the wild-type strain and mutants of *B. cinerea* were inoculated on detached leaves of *Arabidopsis*, one mycelial agar plug per leaf. Photographs were captured at 24 hpi. Bar = 50 μm. (**B**) Stereoscopic observation of ICs formation of wild-type and mutants growing on microscope slides. The mycelial agar plugs (3 mm diameter) of the wild-type strain and mutants of *B. cinerea* were inoculated on glass slides at 20 °C for 24 h, and then examined directly under a stereomicroscope. Bar = 100 μm. (**C**) Microscopic observation of ICs formation of wild-type B05.10 and mutants on onion epidermal cell layers after staining with Trypan blue. Photographs were captured at 24 hpi. Bar = 100 μm.

The *∆Bcser2* and *∆Bcser1*/*2* mutants could infect only wounded leaves of host plants, suggesting that *Bcser2* may affect the formation of infection cushions (ICs) by *B. cinerea* during infection. Therefore, the ICs formation of different strains was investigated according to the previously described protocol with slight modifications [31,32]. Our results showed that the *∆Bcser2* and *∆Bcser1*/*2* strains were unable to form functional ICs on the surface of intact leaves of *Arabidopsis* or glass slides (Figure 5A,B). In contrast, the *∆Bcser1* strain formed complete ICs as did the WT strain on the surface of intact leaves of *Arabidopsis* and glass slides. In addition, we also tested the ICs formation on onion epidermal cell layers, the result showed that all strains could formed ICs, but the ICs formed by *∆Bcser2* and *∆Bcser1*/*2* strains were aberrant and much less than wild type B05.10 strain (Figure 5C). Furthermore, *Bcser2* complementation restored the ability of *∆Bcser2* strains to form ICs (Figure 5). These results indicated that *Bcser2* plays an important role in the formation of ICs, thereby affecting the virulence of *B. cinerea*.

## 3. Discussion

Subtilisin-like serine proteases (also called subtilases) are widespread in many organisms [18,33] and with a broad spectrum of biological functions, and have been gaining increased amounts of attention with regard to their significant role in plant–pathogen interactions [20,22,29,34]. Here, we characterized two genes encoding subtilisin-like proteases (*Bcser1* and *Bcser2*) in *B. cinerea.* Previous studies have shown that *Bcser1* and *Bcser2* had a high level of expression during infection on sunflower [26]. Additionally, their homologous genes in *Sclerotinia sclerotiorum* were also up-regulated at the later stages of infection (24–48 hpi) on *Brassica napus* [35] and were identified as candidate effectors by secretome analysis [36]. We also found that *Bcser1* and *Bcser2* were up-regulated during the sclerotial development stage cultured on PDA plates and the late stage of infection on *Arabidopsis* (Figure 1B,C). Therefore, in this study, we mainly studied the effects of *Bcser1* and *Bcser2* on the sclerotial formation and virulence of *B. cinerea*.

Subtilisin-like serine proteases are reported to be involved in the sporulation of *M. oryzae* and *C. parasitica* [25,34], and impacted the sclerotial development of *V. dahlia* [21]. Our results indicated that *Bcser1* is essential neither for sporulation nor sclerotial formation in *B. cinerea.* In addition, we also analyzed the mycelium tip morphology and acid production of *∆Bcser1,* and there was no obvious difference from the wild type strain B05.10 (Figure S4A,B). In contrast, *∆Bcser2* and *∆Bcser1*/*2* mutants almost completely lost the ability to produce sclerotia on PDA plates. But they can produce small sclerotia on carrots, and the weight is much less compared to wild-type and complementary strains (Figure 2C,D and Figure S5). We speculate that carrots may contain more nutrients or some special ingredients to help the *∆Bcser2* and *∆Bcser1*/*2* mutants form sclerotia. Moreover, both the *∆Bcser2* and *∆Bcser1*/*2* mutants failed to produce any conidia either on PDA plates or on tissues of *Arabidopsis* (Figure 3). In studies of the loss of function of subtilisin-like serine proteases in *M. oryzae* [25,34] and *C. parasitica* [22], the knockout strains still had the ability to produce a small amount of conidia, while the *∆Bcser2* and *∆Bcser1*/*2* mutants of *B. cinerea* completely lost that ability. Our findings indicated that *Bcser2* plays a decisive role in sporulation and sclerotial formation in *B. cinerea.*

Subtilisin-like serine proteases are also reported to play important roles in the virulence of many pathogenic fungi [20]. Orthologous of *Bcser1* and *Bcser2* are widely present in pathogenic fungi, and they also have been reported to play important roles in the virulence of pathogenic fungi [12,21,22,34,37]. For example, disruption of *Spm1,* an orthologous of *Bcser2* in the rice blast fungus *M. oryzae,* resulted in a significant reduction in virulence on seedlings of rice and barley [34]. However, the mechanism by which subtilisin-like serine proteases regulate the virulence of plant pathogens remains unclear. Here, we found that deletion of *Bcser1* did not affect the virulence of *B. cinerea*, *Bcser2* knockout resulted in defective virulence of *B. cinerea* on intact leaves of plants. Both ∆*Bcser2* and ∆*Bcser1*/*2* strains formed obvious lesions on wounded leaves of *Arabidopsis* and tomato (Figure 4), suggesting that *Bcser2* may play a crucial role in the penetration stage during infection by *B. cinerea*. 

Many plant-pathogenic fungi can form a variety of infection structures on the undamaged plant surface, mainly including appressoria and infection cushions (ICs) [31,32,34,38,39,40,41]. These infection structures are important for the direct penetration of host tissue, and functional defects in the infection structures could affect the virulence of plant-pathogenic fungi. For instance, previous studies have shown that disruption of the *Ss-caf1* in *S. sclerotiorum*, resulting in defects in appressorium formation and eventually led to a loss of virulence [40]. In *M. oryzae*, knockout of an important subtilisin-like serine protease-encoding gene, *spm1*, also resulted in a defect in appressorium formation as well as in infectious growth at the post-penetration stage [34]. A number of genes have been reported to be involved in infection structure formation, thereby affecting the full virulence of *B. cinerea* [31,39,42,43,44]. In this study, we found that deletion of *Bcser1* had no effect on the formation of infection cushions in *B. cinerea*. Consistent with the results of the virulence test, both *∆Bcser2* and *∆Bcser1*/*2* mutants completely lost the ability to form infection cushions on the leaves of *Arabidopsis* and on glass slides (Figure 5A,B). In addition, the mutant *∆Bcser2* failed to infect intact leaves of Arabidopsis and tomato, while the complementary strains regained the infection ability (Figure 4A). Furthermore, the mutant *∆Bcser2* could infect wounded leaves of Arabidopsis and tomato, resulting in a lesion that is consistent with the wild type. Therefore, the virulence defects in the *∆Bcser2* and *∆Bcser1*/*2* mutants maybe caused by their inability to form infection cushions during infection. Previous research showed that subtilisin-like serine proteases *spm1* and *prb1* localized in vacuole and play important roles in autophagy, and is also required for the virulence of *M. oryzae* and *C. parasitica* [22,34]. Autophagy is a ubiquitous process for degradation and recycling of the resulting macromolecules, occurring in all eukaryotic cells [45]. Autophagy has been shown to be involved in many life processes of fungi, including cellular differentiation, development, and virulence to host plants [46,47,48,49]. In *B. cinerea*, the autophagy-related genes *BcATG1*, *BcATG4* and *BcATG8* were also involved in the development and virulence [50,51,52]. Interestingly, we found that the phenotype of *∆Bcser2* is very similar to that of these autophagy-related genes in *B. cinerea*. Therefore, we presume that *Bcser2* maybe also involved in autophagy and thus affects the development and virulence of *B. cinerea.*

In summary, we identified two genes, *Bcser1* and *Bcser2*, that encode subtilisin-like serine proteases with an inhibitor I9 domain and a peptidase S8 domain in *B. cinerea.* Our results suggest that *Bcser2* plays important roles in the sclerotial formation, sporulation, infection cushion formation and virulence of *B. cinerea*. Our findings offer crucial clues to help reveal the mechanism of the development and infection process of *B. cinerea*.

## 4. Materials and Methods

### 4.1. Fungal Strains and Growth Conditions

The *Botrytis cinerea* wild-type strain B05.10 and its derived mutant strains were used in this study. All strains were routinely cultured on potato dextrose agar (PDA, 200 g potato, 20 g glucose, 20 g agar and 1 L water). The B05.10 strain was maintained in PDA slants at 4 °C for further use. Knock-out mutants were maintained on PDA amended with 75 μg/mL hygromycin B (Calbiochem, San Diego, CA, USA) or 75 μg/mL G418 (Sigma-Aldrich, St. Louis, MO, USA). Complementation mutants were maintained on PDA amended with 75 μg/mL hygromycin. For growth experiments, the mutants and B05.10 were grown on PDA at 20 °C. Each plate was inoculated with a 5-mm-diameter mycelial agar plug taken from the edge of a 2-day-old colony. To characterize the growth rate, sclerotia formation and infection cushion formation, different strains were cultured in constant darkness. To characterize the sporulation, strains were grown under a 12 h light/dark cycles. Each experiment was repeated three times independently.

### 4.2. Bioinformatics Analysis

The homology analysis was based on BlastP at the National Center for Biotechnology Information (NCBI) website (http://blast.ncbi.nlm.nih.gov/Blast.cgi), and the amino acid sequence of SPM1 (MGG_03670) in the rice blast fungus *Magnaporthe oryzae* was used as a query sequence. The *Bcser1* and *Bcser2* genes were characterized using the publicly available genomic sequence database of *B. cinerea* (http://fungi.ensembl.org/Botrytis_cinerea). SignalP 4.0 was used for signal peptide prediction [53].

### 4.3. Gene Replacement and Complementation Strategy

A split-marker system was used for the targeted gene deletion of *Bcser1* and *Bcser2* in *B. cinerea* [54]. The disruption strategy for *Bcser1* and *Bcser2* is outlined in Supporting Information Figure S1. For the deletion of *Bcser1*, the 5′ flank (603 bp) and the 3′ flank (635 bp) of the *Bcser1* ORF were amplified from the genomic DNA of *B. cinerea* B05.10 using PrimeSTAR HS DNA Polymerase (Takara, Shiga, Japan) with primers P1/P2 (containing a *Kpn*I/*Xba*I site) and P3/P4 (containing a *Sal*I/*Hind*III site), respectively (Table S1). As a selection marker, the hygromycin resistance gene (*hph*) cassette from the vector pUCH18 [55] was used. The two fragments were then respectively cloned into pUCH18 to construct pUCH18-Bcser1-5′ and pUCH18-Bcser1-3′. The two fused fragments, Bcser1-5′-HY and YG-Bcser1-3′ were amplified with primers P1/HY and YG/P4, respectively (Table S1). The neomycin resistance gene (*neo*) cassette from the vector pCETNS was used as a selection marker for *Bcser2* deletion, and *∆Bcser1* was used as a recipient strain for the *Bcser1* and *Bcser2* double deletion. The 5′ flank (732 bp) and the 3′ flank (778 bp) of the *Bcser2* ORF were amplified from the genomic DNA of *B. cinerea* B05.10 with primers P5/P6 and P7/P8, respectively (Table S1). The overlapping marker fragments “NE” and “EO” of the *neo* cassette were amplified from pCETNS using the P9/P10 and P11/P12 primers, respectively (Table S1). The 5′ extensions of primers P6 and P7, facilitating fusion of the gene flanks with the truncated marker fragments, are complementary to the P9 and P11 primer sequences, respectively. The fused fragments *Bcser2*-5′-NE and EO-*Bcser2*-3′ were amplified by two round fusion PCR as described by Catlett (2003), with primers P5/P10 and P11/P8, respectively.

To indicate that the phenotype of *∆Bcser2* is due to the deletion of the *Bcser2* gene, an in situ complementation assay was performed to rescue the phenotype. The complementation strategy of *Bcser2* is illustrated in Figure S2. First, the predicted full-length *Bcser2* gene with 5′ flank (including promoter and coding sequence, 3158 bp) and 3′ flank (778 bp) of the *Bcser2* ORF were amplified from genomic DNA of *B. cinerea* strain B05.10 using primers P1/P13 and P14/P4, respectively (Table S1). To replace the *neo* cassette selection marker, the overlapping marker fragments “HY” and “YG” of the *hph* cassette are amplified from pUCH18 [55] using the HYG-F/HY-R and YH-F/HYG-R primers, respectively (Table S1). The 5′ extensions of primers P13 and P14, facilitating fusion of the gene flanks with the truncated marker fragments, are complementary to the HYG-F and HYG-R primer sequences, respectively. As mentioned before, the fused fragments 5′-*Bcser2*-HY and YG-*Bcser2*-3′ were amplified by a two-round fusion PCR as described by Catlett (2003), with primers P1/HY-R and YG-F/P4, respectively. The purified fragments were used for PEG-mediated protoplast transformation.

### 4.4. Transformation of B. cinerea

*B. cinerea* wild-type strain B05.10 was used for transformation experiments. Transformation of *B. cinerea* was performed according to the previously described protocol with slight modifications [56,57].

For preparing protoplasts, *B. cinerea* strain B05.10 (or *∆Bcser1*, used for *Bcser2* gene deletion, or *∆Bcser2*, used for *Bcser2* gene complementation) was grown on PDA plates covered with cellophane membranes at 20 °C. Young mycelium was harvested in a clean bench with a sterilized inoculation needle at 2 days post inoculation (dpi) and then transferred into a sterilized flask (50 mL) containing 10 mL cell wall lysis solution (0.1% snailases (Dingguo, Beijing, China), 1% lysis enzymes (Sigma-Aldrich), dissolved in osmosis stabilizing buffer (0.6 M KCl, 50 mM CaCl_2_)) per gram of fresh weight [55]. The mycelium suspension was incubated in a shaker for 2 h at 28 °C with shaking at 120 rpm. The digested suspension was filtered through four-layer lens wiping paper and pelleted by centrifugation at 8000 g for 5 min. Protoplasts were then washed once and resuspended in osmosis stabilizing buffer (0.6 M KCl, 50 mM CaCl_2_) for a final concentration of 1 × 10^7^ protoplasts/mL. 

For PEG mediated transformation of *B. cinerea* protoplast, first, 100 μL of the protoplast suspension was maintained on ice for 5 min. Second, two split-marker DNA fragments purified previously (10 μg each), 5 μL of 50 mM spermidine (Sigma) and 100 μL of 40% (wt/vol) PEG 3350 (Sigma) in 0.6 M KCl, 50 mM CaCl_2_, and 50 mM Tris-HCl (pH 7.5) were gently added to the protoplasts in turn and mixed well, followed by incubation on ice for 20 min. Third, 750 μL of the same PEG solution was added to the mixture and incubated at room temperature for 10 min. Finally, this transformation mixture was spread on plates (200 μL per plate) containing RM agar medium (0.05% yeast extract, 0.7 M sucrose and 1% agar), and incubated for 16–20 h at 20 °C to regenerate the mycelium. The next day, regenerated mycelium was overlaid with RM agar medium supplemented with hygromycin B or G418 (100 μg/mL). After 3–10 days of incubation at 20 °C, drug-resistant colonies of *B. cinerea* appeared on the surface of the selection medium. Hyphal tips of resistant colonies were transferred to PDA plates containing hygromycin B or G418 (75 μg/mL), followed by at least three rounds of hyphal tip isolation.

Homokaryotic strains of transformants were obtained by single spore isolation which was performed following a previously described protocol with some modifications [58,59]. Conidia of heterokaryotic transformants developed on PDA plates containing hygromycin B or G418 (75 μg/mL) were harvested and then diluted with sterile water, and 20 to 50 spores were spread on selection medium supplemented with hygromycin B or G418 (100 μg/mL). Subsequently, germlings from a single conidium were isolated after 48 h and transferred to a new selection medium. 

### 4.5. Molecular Analysis of B. cinerea Mutants

Initial screening of the transformants was performed by PCR, and the validation strategy is illustrated in Figure S1. The primers used for PCR validation of *Bcser1* (P15/HY-R, YG-F/P16, HYG-F/HYG-R and P17/P18), *Bcser2* and *Bcser1*/*2* (P19/NE-R, EO-F/P20, NEO-F/NEO-R and P21/P22), and *Bcser2-C* (P19/HY-R, YG-F/P20, HYG-F/HYG-R and P21/P22) mutants are listed in Table S1).

To further confirm that the targeted gene was replaced by a single copy marker gene, a Southern blot was performed as described by Zhang (2016). Genomic DNA of *B. cinerea* was extracted from young mycelium with the CTAB method [55], and 15–20 µg DNA from each strain was digested with *EcoR*V. Hybridization analysis was performed using the Amersham AlkPhos Direct Labelling and Detection Systems (GE Healthcare, Piscataway, NJ, USA). Probes were amplified from *hph* and *neo* with primers P23/P24 and P25/P26, respectively (Figure S1; Table S1) and labelled with alkaline phosphatase.

### 4.6. RNA Isolation and qRT-PCR

Total RNA samples of *B. cinerea* were isolated using the RNAiso Plus regent (Takara) according to the manufacturer’s instructions and stored at minus 80 degrees for further study. The RNase-free Recombinant DNase I (Takara) treatment was performed to eliminate residual genomic DNA, and the M-MLV Reverse Transcriptase (Promega, Madison, WI, USA was used to generate the first-strand cDNA.

Quantitative real-time reverse transcriptase PCR (qRT-PCR) was performed by the using of CFX96 Real-Time PCR Detection System (Bio-Rad, Hercules, CA, USA with iTaq universal SYBR Green supermix (Bio-Rad), according to the manufacturer’s instructions. The *B. cinerea Actin* gene (Bcin16g02020) was used as reference gene for normalizing the RNA samples [26,60]. Primer were designed across or flanking an intron (Table S1). For each gene detecting, qRT-PCR assays were repeated at least twice and each with three independent biological replicates.

### 4.7. Virulence Assay

The *A. thaliana* and tomato (*Lycopersicum esculentum*) plants used in these experiments were grown in a climate chamber at 20 °C with a 16-h photoperiod. Virulence assays for the characterization of *B. cinerea* strains (wild-type and mutants) were performed on detached leaves from 5-week-old *A. thaliana* and tomato. Because the mutants (*∆Bcser2* and *∆Bcser1*/*2*) lost their ability to produce spores, we inoculated the plants with mycelium pellets. Leaves were inoculated with mycelial agar plugs (diameter = 3–4 mm) from the margins of colonies actively growing on PDA. For injury tests, each leaf was wounded by a sterilized inoculation needle to make three small holes, and a mycelial agar plug was inoculated onto the wound. All of the inoculated leaves were placed in a plastic box lined with absorbent paper moistened with sterile water. The storage box was sealed with transparent plastic film to maintain high humidity and then incubated in a climate chamber at 20 °C with a 16-h photoperiod. Virulence was evaluated by lesion diameters that were measured at 48 hpi for *Arabidopsis* or at 72 hpi for tomato [61]. Six leaves from three plants (two leaves per plant) were used for each treatment. Experiments were repeated at least three times.

### 4.8. Stereomicroscopic Observation of Infection Cushions

Stereomicroscopic observation of infection cushions was performed according to a previously described method with modifications [31,32]. The mycelial agar plugs (3 mm diameter) of wild-type strain B05.10 and mutants of *B. cinerea* were inoculated on microscope slides or on leaves of *Arabidopsis* with one mycelial agar plug per microscope slide or leaf, three scales for each strain. All inoculated microscope slides or leaves were placed on moistened absorbent paper in a plastic box and covered with transparent plastic film to maintain high humidity. After incubation of the leaves at 20 °C for 24 h, the mycelial and the leaves of *Arabidopsis* were stained with trypan blue and examined for the formation of ICs under a stereomicroscope. The incubated of microscope slides were examined directly under a stereomicroscope. The number of ICs formed by each *B. cinerea* strain around the mycelial agar plugs was counted.

### 4.9. Statistical Analysis

Data significance were analyzed using analysis of variance carried out with SAS version 8.1 (SAS Institute, Cary, NC, USA) ANOVA. When significant treatment differences were found, treatment means were separated by the protected least significant difference test at *p* ≤ 0.01. Different letters or asterisks in the graphs indicate statistical differences.

## Supplementary Materials

Supplementary materials can be found at https://www.mdpi.com/1422-0067/21/2/603/s1.
