# Supplementary material for: The Subtilisin-Like Protease Bcser2 Affects the Sclerotial Formation, Conidiation and Virulence of Botrytis cinerea
Source: Int J Mol Sci. 2020 Jan 17;21(2):603. doi: 10.3390/ijms21020603 (PMC7013506; doi:10.3390/ijms21020603)
Supplement: Supplementary file 1 [file ijms-21-00603-s001.zip › ijms-682171-supplementary-orinigal/ijms-682171-supplementary-orinigal.pdf]

Supplementary:

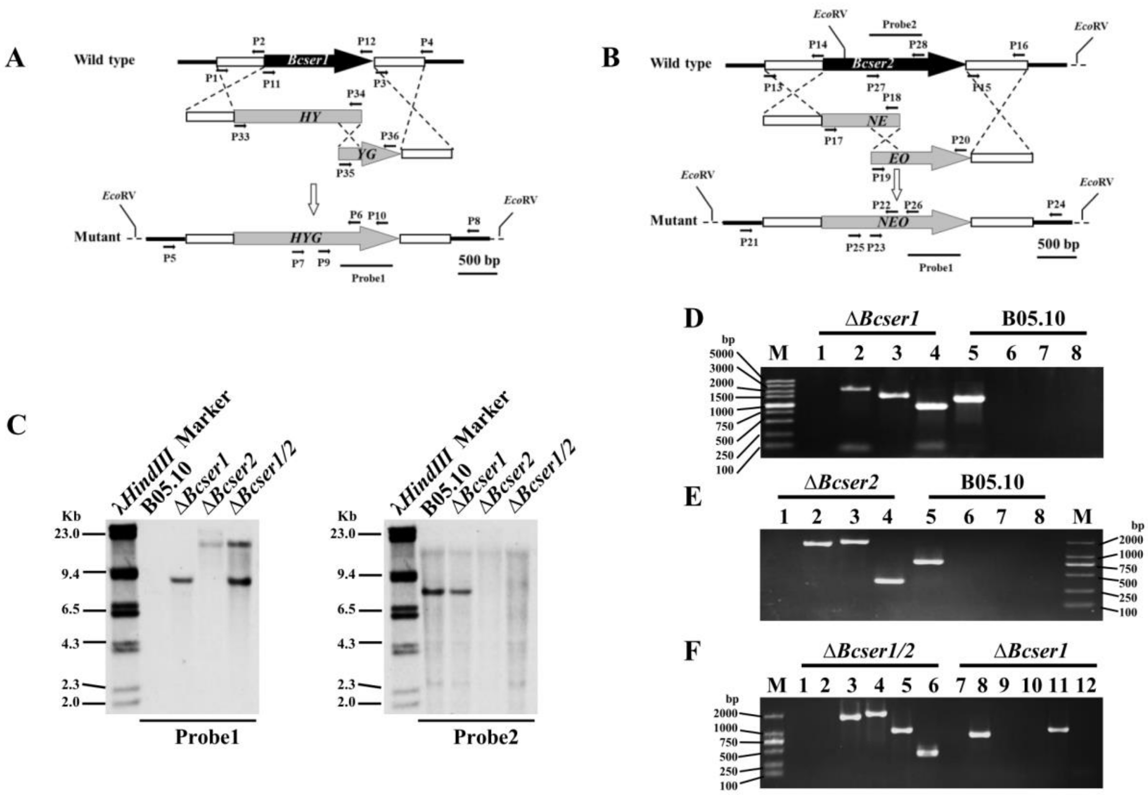

Figure S1. Construction and identification of *Bcser1* and *Bcser2* deletion mutants. (**A**) Schematic diagram of the *Bcser1* deletion strategy, the different shading shows the *HptII* cassette (gray box), *Bcser1* (black box) and flanked sequences (white box). The scheme was drawn to scale according to sequence length. (**B**) Schematic diagram of the *Bcser2* deletion strategy, the different shading shows the *G418* cassette (gray box), *Bcser2* (black box) and flanked sequences (white box). The scheme was drawn to scale according to sequence length. (**C**) Southern blot hybridization analysis of deletion mutant Δ*Bcser1*, Δ*Bcser2*, Δ*Bcser1/2* and wild-type strain B05.10. Genomic DNA from all strains was digested by *Eco*RV. Probe 1 (from the trpC terminator) was amplified with primer pair P1/P2, and probe 2 (from the *Bcser2* gene) was amplified with primer pair P27/P28. Probes were labelled with alkaline phosphatase according to the manual. (**D**) PCR validation of the *Bcser1* deletion mutant. The full length fragment of the *Bcser1* gene was amplified with primer pair P11/P12 (lanes 1, 5); the upstream homologous recombination of *Bcser1* was validated by primer pair P5/P6 (lanes 2, 6); the downstream homologous recombination of *Bcser1* was validated by primer pair P7/P8 (lanes 3, 7); the *HptII* homologous recombination was validated by primer pair P9/P10 (lanes 4, 8). M, DL5000 Marker. (**E**) PCR validation of the *Bcser2* deletion mutant. The validation strategy is the same as (**D**), and the primer pairs used were P27/P28 (lanes 1, 5), P21/P22 (lanes 2, 6), P23/P24 (lanes 3, 7), and P19/P18 (lanes 4, 8). M, DL2000 Marker. (F) PCR validation of the *Bcser1* and *Bcser2* double deletion mutants. The gene fragments of *Bcser1* and *Bcser1* were amplified with primer pairs P11/P12 (lanes 1, 7) and P27/P28 (lanes 2, 8), respectively; the upstream and downstream homologous recombination fragments of *Bcser2* were amplified with primer pairs P27/P28 (lanes 3, 9) and P21/22 (lanes 4, 10), respectively; *HptII* and G418 homologous recombination fragments were amplified with primer pairs P9/P10 (lane 5, 11) and P19/P18 (lane 6, 12), respectively. M, DL2000 Marker

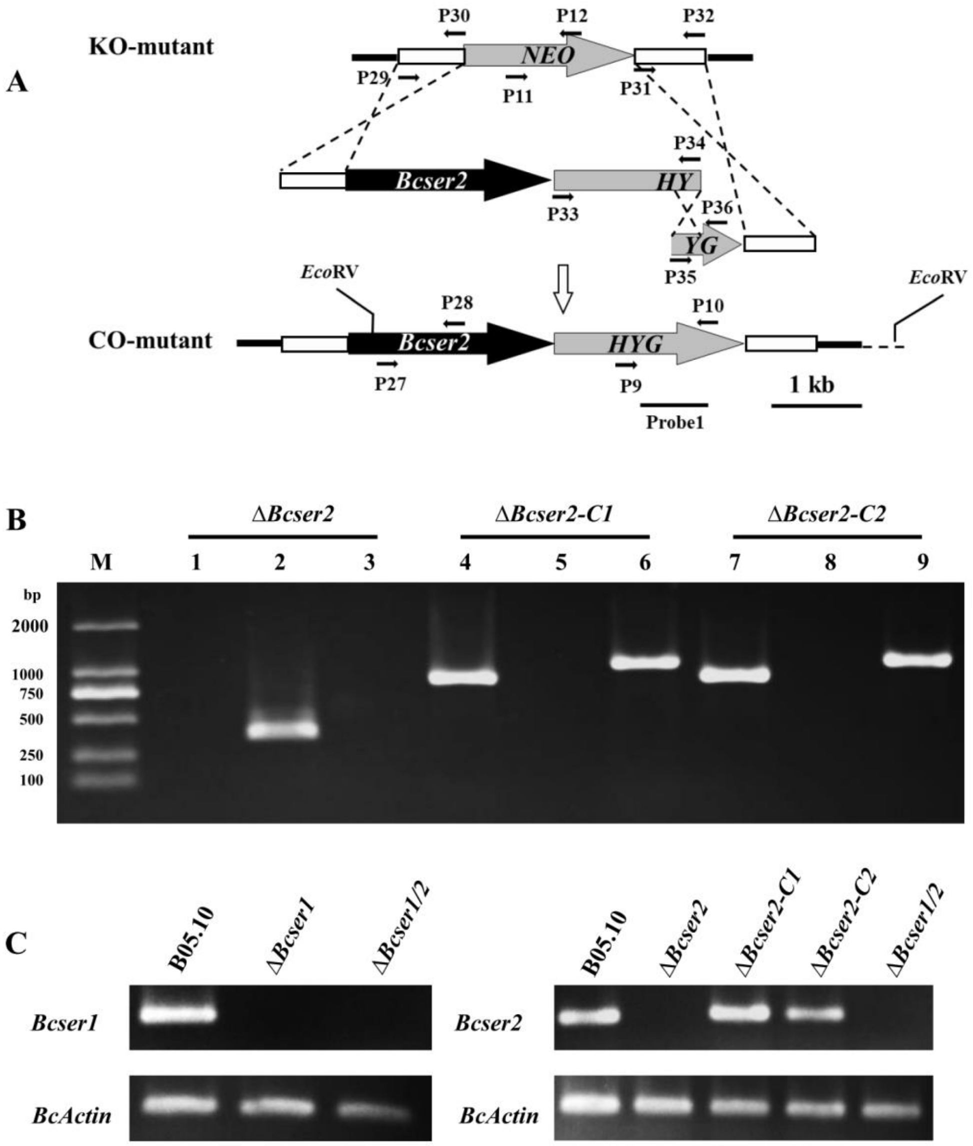

Figure S2. Construction and identification of *Bcser2* complementary mutants. (**A**) Schematic diagram of the *Bcser2* complementation strategy. An in situ complementation method was used for *Bcser2* complement, and Δ*Bcser1* was used as the original strain. The scheme was drawn to scale according to sequence length. (**B**) PCR validation of *Bcser2* complementary mutants. The gene fragment of *Bcser2* was amplified with primer pair P27/P28 (lane 1, 4, 7); the G418 fragment was amplified by primer pair P19/P18 (lane 2, 5, 8); the homologous recombination fragment of *HptII* was amplified by primer pair P9/P10 (lane 3, 6, 9). M, DL2000 Marker. (**C**) RT-PCR analysis of the transcription of *Bcser1* and *Bcser2* in wild type B05.10 and corresponding mutants with gene specific primer pairs P37/P38 and P39/P40 (Table S1), respectively. The *BcActin* gene in *B. cinerea* was used as the reference gene.

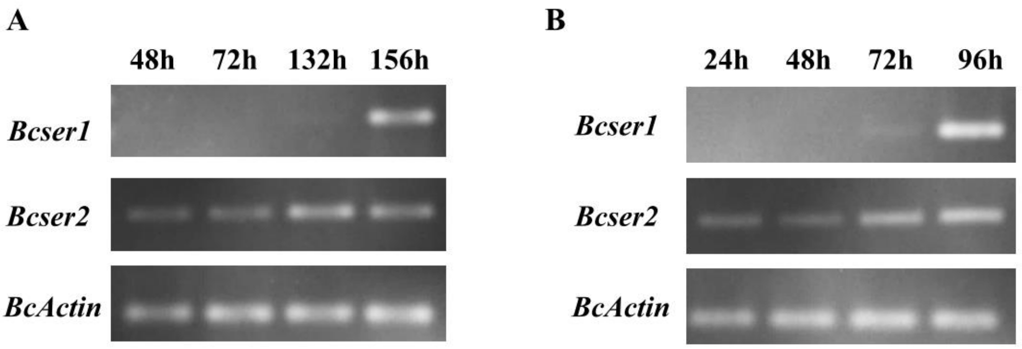

Figure S3. RT-PCR analysis showing the expression patterns of *Bcser1* and *Bcser2* in B05.10 strain. (**A**) Expression patterns of *Bcser1* and *Bcser2* when cultured on PDA medium at 20℃. (**B**) Expression patterns of *Bcser1* and *Bcser2* when inoculated on *Arabidopsis* plants at 20 ℃.

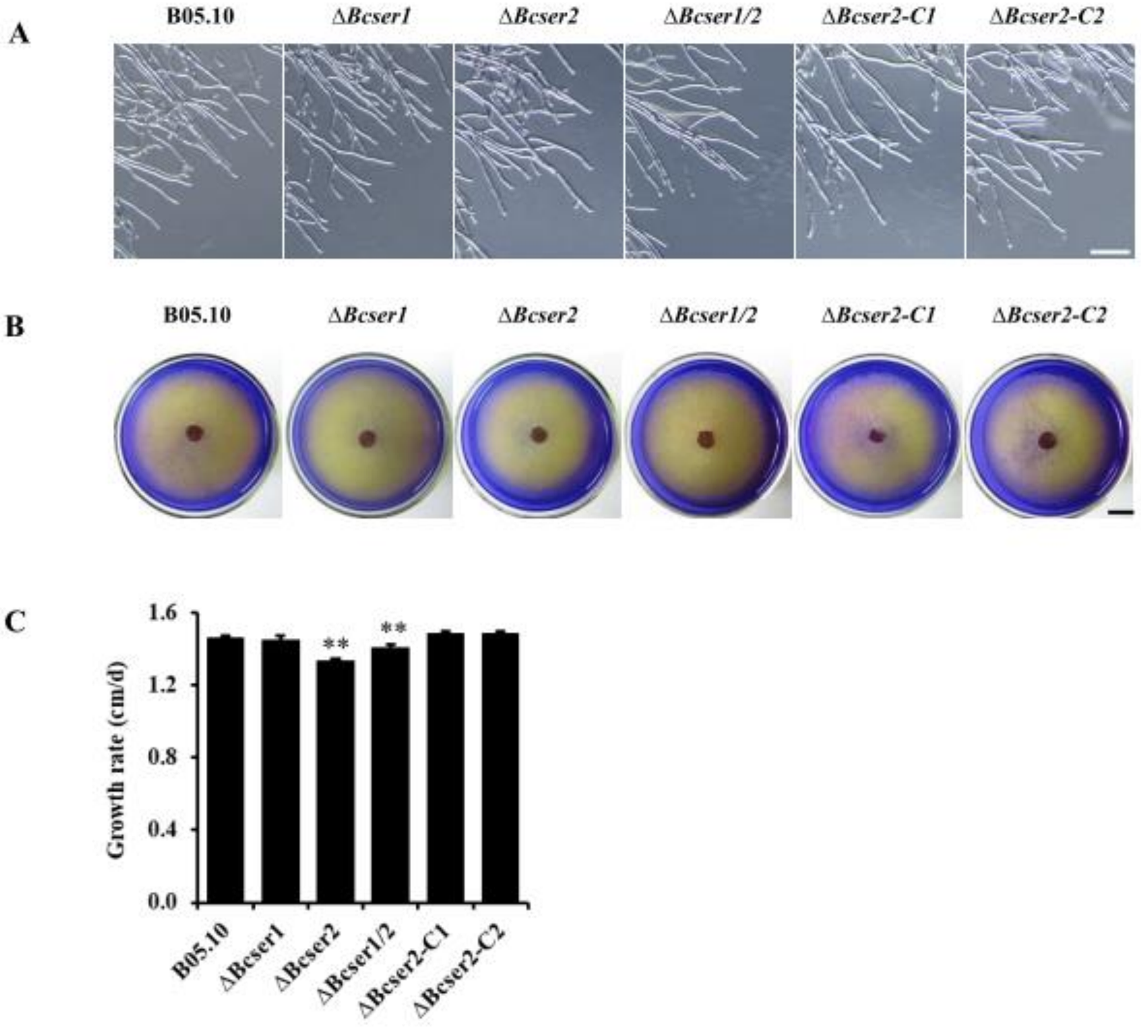

Figure S4. Determination of the biological characteristics of *B. cinerea*. (**A**) Hyphal tip

growth and branching patterns of deletion mutants Δ*Bcser1*, Δ*Bcser2* and Δ*Bcser1/2*, complemented mutant Δ*Bcser2-C1* and Δ*Bcser2-C2*, and wild-type B05.10 grown on PDA plate covered with cellophane. Scale bar, 100 μm. (**B**) Qualitative detection of acid produced by deletion mutants Δ*Bcser1*, Δ*Bcser2* and Δ*Bcser1/2*, complemented mutants Δ*Bcser2-C1* and Δ*Bcser2-C2*, and wild type B05.10 on PDA plates containing 0.005％ (w/v) bromophenol blue dye as a pH indicator. The media color turning from blue to yellow indicates that acid was produced. Photographs were captured at 48 hpi. Scale bar, 1 cm. (**C**) Growth rate of strains growing on a PDA plate at 20℃ in complete darkness. Three independent replications were performed for each treatment. Bars indicate ±SE. Statistical significance is indicated in the graph (one-way ANOVA): **, $P < 0.01$.

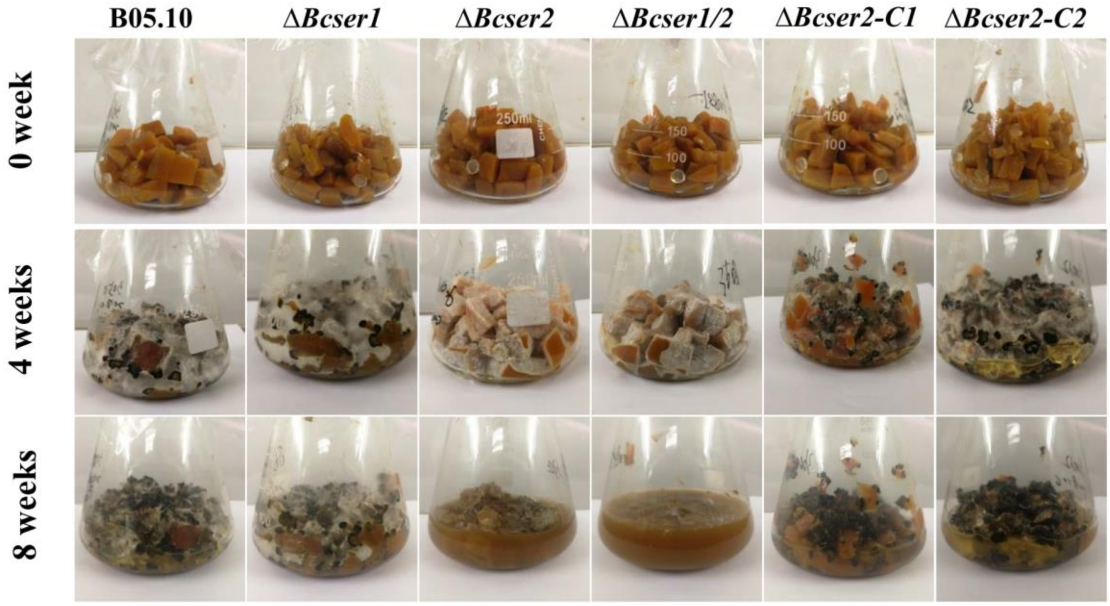

Figure S5. Inoculation of wild-type strain B05.10 and all mutants into sterilized carrot cube medium in flasks. Photographs were captured at 0, 4, and 8 weeks post-inoculation, respectively.

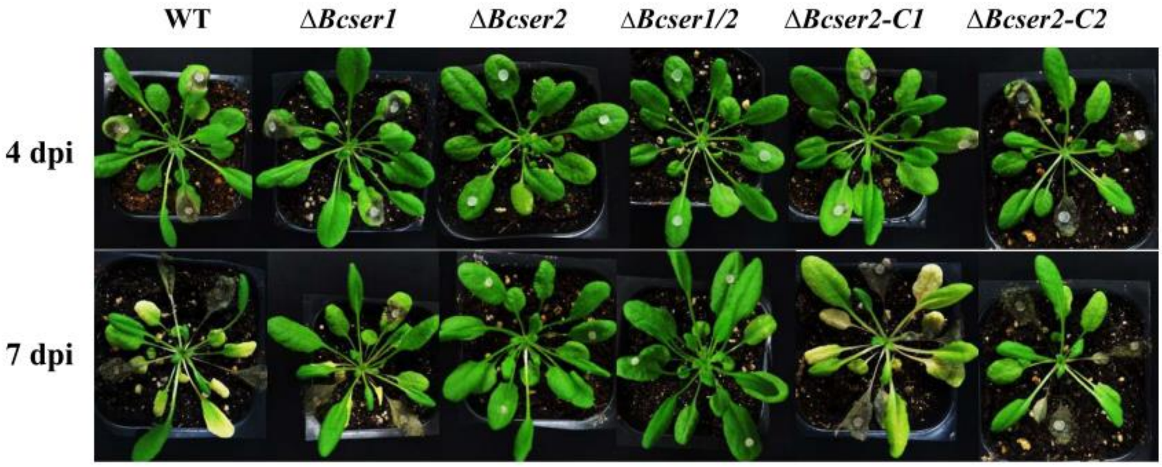

Figure S6. Virulence assays of mutants and wild-type strains on detached *Arabidopsis*. Photographs were captured at 4 dpi and 7 dpi.
